# Cerebellar Dentate Cavernoma Enlargement and Tremor Emergence: Longitudinal Neuroimaging Analysis of Case Report and Literature Review

**DOI:** 10.1002/mdc3.70130

**Published:** 2025-05-19

**Authors:** Andrew Z. Yang, Tiffany Rodrigues, Sriranga Kashyap, Yash Patel, Arjun Balachandar, Benjamin A. Davidson, Can Sarica, Rafael E. Buongermini, Artur Vetkas, Abu Bakar Butt, Jurgen Germann, Alfonso Fasano, Suneil K. Kalia, Andres M. Lozano, Marie Slegr, Alexandre Boutet

**Affiliations:** ^1^ Division of Neurosurgery, Department of Surgery University of Toronto Toronto Ontario Canada; ^2^ Joint Department of Medical Imaging University of Toronto Toronto Ontario Canada; ^3^ Krembil Brain Institute, Toronto Western Hospital (TWH) University Health Network (UHN) Toronto Ontario Canada; ^4^ Center for Advancing Neurotechnological Innovation to Application (CRANIA) Toronto Ontario Canada; ^5^ Institute of Medical Science, University of Toronto Toronto Ontario Canada; ^6^ Edmond J. Safra Program in Parkinson's Disease, Morton and Gloria Shulman Movement Disorders Clinic, TWH, UHN Toronto Ontario Canada; ^7^ Sunnybrook Research Institute Toronto Ontario Canada; ^8^ Harquail Centre for Neuromodulation, Hurvitz Brain Sciences Program, Sunnybrook Research Institute Toronto Ontario Canada; ^9^ Schulich School of Medicine and Dentistry Western University London Ontario Canada; ^10^ Division of Neurology University of Toronto Toronto Ontario Canada; ^11^ KITE Research Institute, UHN Toronto Ontario Canada; ^12^ Division of Neurology Neurologist Toronto Ontario Canada

**Keywords:** cavernoma, cerebellum, dentate, tractography, tremor

Cavernoma, cavernous angioma, or cerebral cavernous malformation, is a central nervous system vascular pathology.^1^ These acquired lesions are typically asymptomatic, but can cause seizures, headaches, hemorrhage, and, infrequently, tremors.^1^ This study reports an atypical unilateral action and rest tremor emerging concurrently with a cavernoma in the ipsilateral cerebellar dentate nucleus (DN). The hypothesis is that lesion growth disrupts the motor pathway dentato‐rubro‐thalamic tract (DRTT), precipitating tremor onset. The study aims to (1) quantify cavernoma volume change; (2) examine lesion increase in relation to neurocircuitry; and (3) compare findings to a literature review of tremors and cavernomas.

## Case Report

A 60‐year‐old right‐handed woman presented with a right‐hand tremor that emerged 4 months prior. History included a transient episode of right arm heaviness with tingling and an episode of a painless visual aura of migraine 16 years prior. Her 88‐year‐old father was described as having “shaky” hands but otherwise healthy. Magnetic resonance imaging (MRI) demonstrated a right DN cavernoma, which was first identified incidentally 16 years ago, where she was followed conservatively by her vascular surgeon and received routine MRIs in 2008, 2016, and 2018. When tremor emerged in 2024, her neurologist ordered an MRI. Neurological examination showed a mild right‐handed intermittent tremor, more predominant with action, but also present at rest. The tremor reportedly worsened when anxious or when excess caffeine is consumed. Negatives for essential tremor included no bilaterality of symptoms and ataxia, whereas for parkinsonism included no bradykinesia, rigidity, and constipation, as well as no changes in voice, facial expression, writing, manual dexterity, muscle tone, or gait. For management, she chose monitoring rather than medication or surgical treatment.

**FIG. 1:**
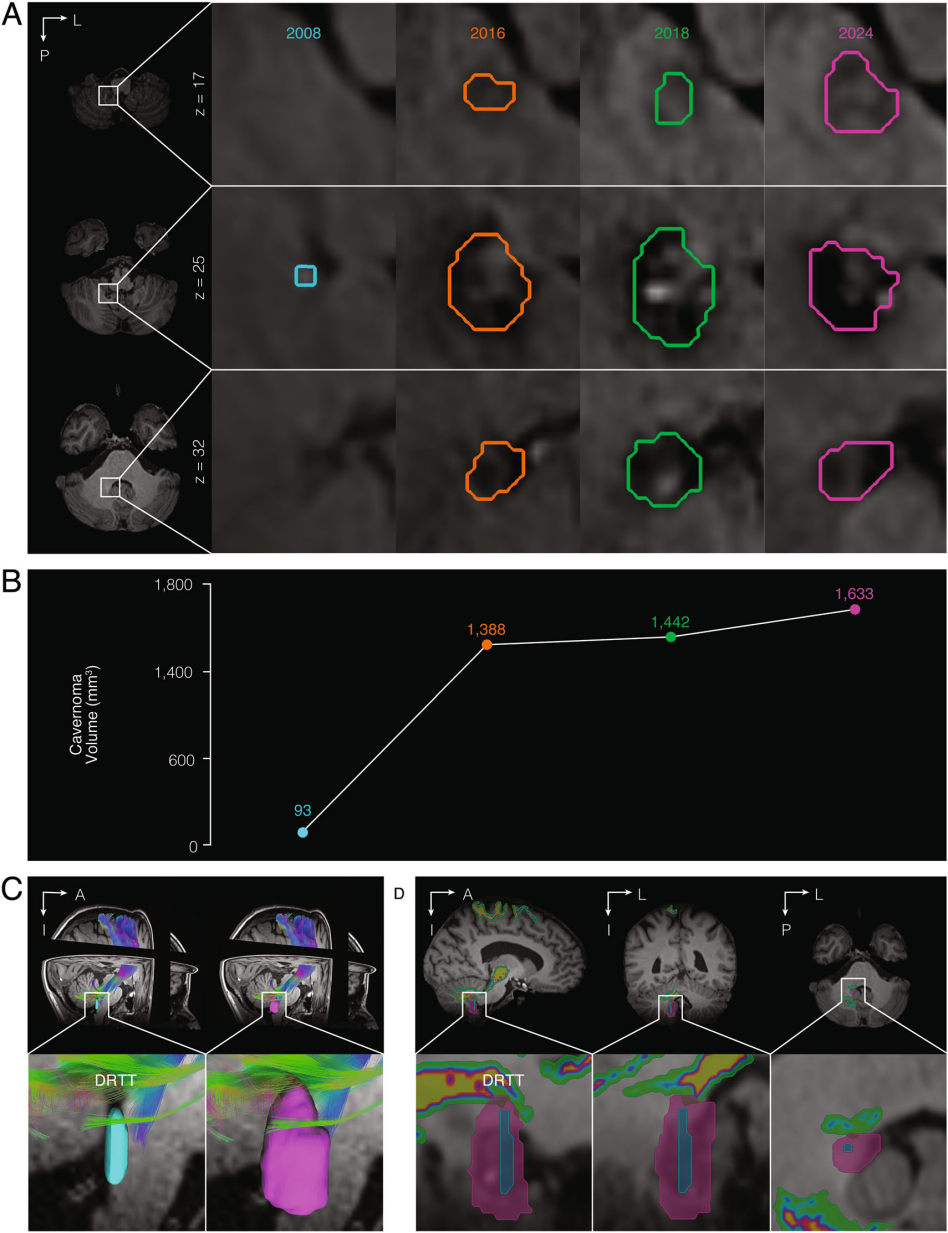
Neuroimaging analysis of cavernoma growth over time. (**A**) T2 fluid‐attenuated inversion recover magnetic resonance imaging (MRI) of the patient's cavernoma with three axial slices (z = *17*, z = *25*, and z = 32) for each neuroimaging time point: 2008 (cyan), 2016 (orange), 2018 (green), and 2024 (magenta) with corresponding outline of the lesion segmentation boundary. (**B**) Line graph of the cavernoma volume over time, highlighting the expansion from 93 mm^3^ in 2008 (cyan), 1388 mm^3^ in 2016 (orange), 1442 mm^3^ in 2018 (green), and 1633 mm^3^ in 2024 (magenta). (**C**) Tractography results demonstrating the patient's dentato‐rubro‐thalamic tract (DRTT), as well as three dimensional reconstructions of the cavernoma segmentations from 2008 (cyan) and 2024 (magenta), which are overlaid on the patient's most recent T1 MRI, indicating increased lesion size over time with increased proximity to the DRTT. (**D**) T1 MRI images in sagittal, coronal, and axial slices reveal apparent cavernoma impingement of the DRTT (color spectrum) in 2024 (magenta) but not in 2008 (cyan). A, anterior; DRTT, dentato‐rubro‐thalamic; I, inferior; L, left; P, posterior.

**FIG. 2:**
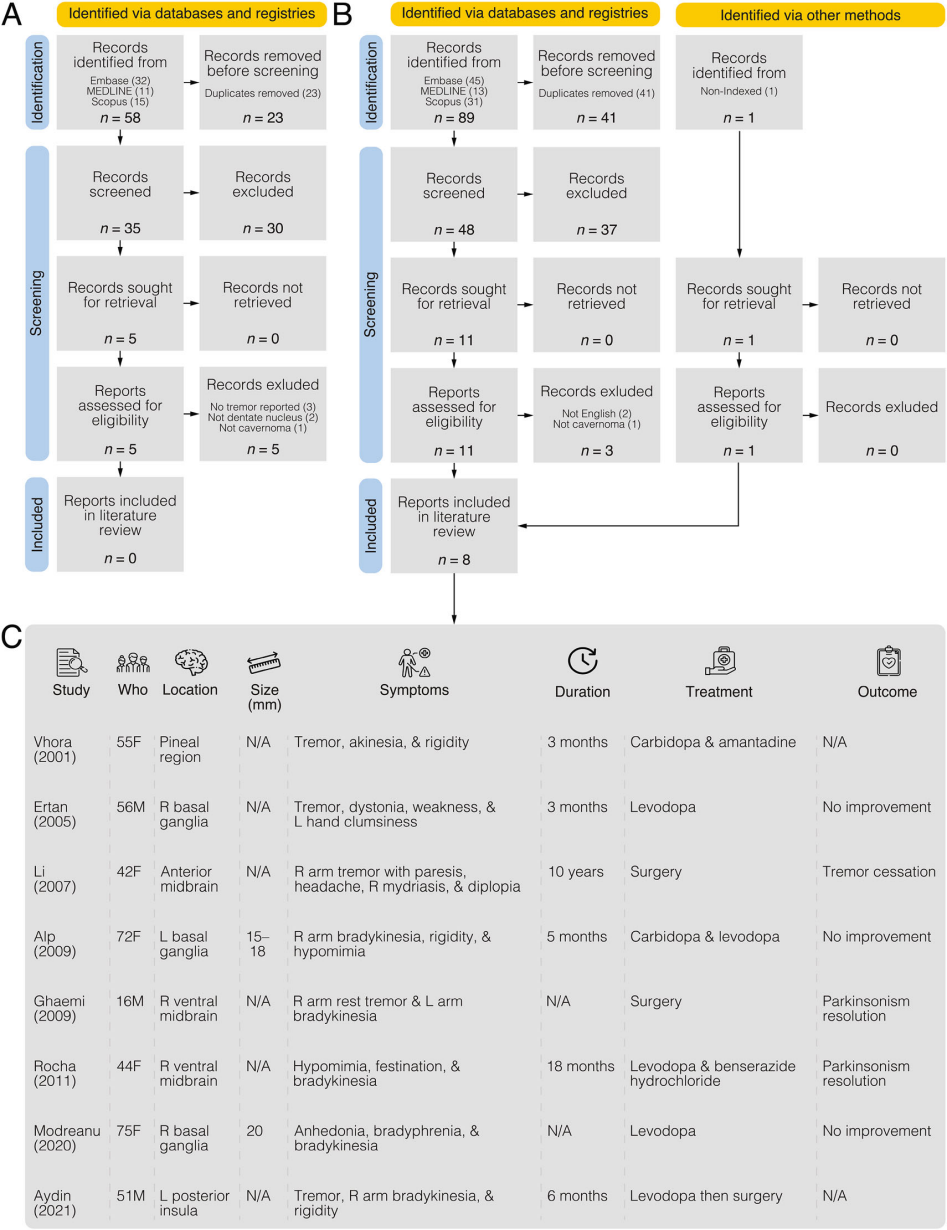
Literature review of cavernoma and tremor. (**A**) Preferred Reporting Items for Systematic reviews and Meta‐Analyses (PRISMA) flow diagram for the preliminary literature review to identify cases of (1) tremor associated with (2) cavernoma localized to the (3) dentate nucleus of the cerebellum. Databases were queried from their inception to July 3, 2024, which included Embase (n = 32), MEDLINE (n = 11), and Scopus (n = 15), resulting in 58 articles. After removing duplicates (n = 23), 35 records underwent abstract screening, leading to full text screening of five reports, but yielded no relevant articles fitting our search criteria. (**B**) PRISMA flow diagram of a subsequent literature search of (1) parkinsonism and (2) cavernoma. Databases were queried from their inception to July 3, 2024, which included Embase (n = 45), MEDLINE (n = 13), and Scopus (n = 31), resulting in 89 articles. After removing duplicates (n = 41), 48 records underwent abstract screening, leading to full text screening of 11 reports, resulting in the inclusion of eight articles for the final analysis. (**C**) Results of articles regarding cavernoma and parkinsonism, including study first author, patient age/sex, cavernoma brain location, symptoms, clinical presentation duration, treatment and follow‐up outcomes. F, female; L, left; M, male; R, right.

The methodology (see Data S1) involved segmenting cavernoma MRIs from 2008, 2016, 2018, and 2024, (Fig. 1A), quantifying volumes (Fig. 1B), tractography to examine lesion relation to the DRTT (Fig. 1C), and a literature search regarding cavernomas and tremors (Fig. 2A,B). Results revealed progressive lesion growth: 93 mm^3^ in 2008, 1388 mm^3^ in 2016, 1442 mm^3^ in 2018, and 1633 mm^3^ in 2024 (Fig. 1A,B). Tractography provided patient‐specific DRTT, which, when compared to longitudinal cavernoma segmentations, indicated recent lesion impingement in 2024 (Fig. 1C,D). The literature review yielded no publications of tremor caused by cerebellar DN cavernoma (Fig. 2A). A subsequent search identified eight articles of secondary parkinsonism due to cavernoma (Fig. 2B,C). Locations were the basal ganglia (n = 3/8), ventral mesencephalon (n = 3/8), insula (n = 1/8), and pineal region (n = 1/8). Median age was 53 (5 female/3 male), median tremor duration was 3 months, and diameters spanned 10 to 20 mm. Dopaminergic treatment for five of eight patients led to one improvement, while three of eight received surgery with two improvements.

## Discussion

The present study reports the atypical emergence of tremor coinciding with cavernoma enlargement revealed via longitudinal neuroimaging analysis. This is unique given the cavernoma location in the DN, its growth disrupting the DRTT, and its temporal association with the notable presence of both rest and action tremor. The quantitative volume change is noteworthy as the visual radiological interpretation reported the lesion as stable.^2^ Potential mechanisms include mass effect, hemorrhage, neuroinflammatory response, or ischemia.^3^, ^4^

The literature review of cavernomas in the cerebellar DN causing tremors yielded no articles, highlighting the case's rarity. The DN, a critical hub for cerebellar output, integrates motor and cognitive functions through its projections along the DRTT, facilitating cerebello‐thalamo‐cortical communication essential for movement and high‐order cognitive processes.^4^ Other types of DN lesions can cause motor and non‐motor symptoms depending on location, such as action tremor (but not rest), ataxia, dystonia, and cognitive impairments.^4^, ^5^ Tremors ensuing from cerebellar lesions include Holmes tremor, previously known as rubral tremor, and cerebellar outflow tremor.^6^ Holmes tremor is often irregular, of high amplitude, and present at rest, intensifying with action and posture.^7^ However, Holmes tremor usually localizes to the brainstem or diencephalon.^7^ Alternatively, parkinsonism is worth considering, given the DRTT implicated in its pathophysiology.^8^ Yet, although the patient presented with unilateral rest tremor, which is typical of parkinsonism, her action tremor and lack of bradykinesia questions this diagnosis. Although the eight literature cases support the causative relation of cavernoma and parkinsonism, the lesions were in regions other than the cerebellar DN, complicating diagnosis.

According to tremor guidelines, “indeterminate tremor syndrome” is appropriate and further monitoring is required for diagnosis, which corresponds with the patient's preferred management plan.^9^ Cavernoma treatment varies according to size, patient preference, symptom severity, and location.^1^ The first treatment consideration is often open cranial surgery with promising evidence for stereotactic techniques (reasonable outcomes of cerebellar cavernoma surgery was reported as 129/141 = 91%).^10^ Further research is required to elucidate the relationship between cavernoma in the DN and tremor, and this study has limitations, namely that it is a single case report with manual segmentations. Overall, this study reports the unusual emergence of action and rest tremor potentially because of an enlarging cavernoma in the DN now impinging on the DRTT, which may guide future diagnoses and prevent misdiagnoses.

## Author Roles

(1) Research project: A. Conception, B. Organization, C. Execution. (2) Manuscript Preparation: A. Writing of the First Draft, B. Review and Critique.

A. Z. Y.: 1A, 1B, 1C, 2A, 2B

T. R.: 1A, 1B, 1C, 2A, 2B

S. K.: 1C, 2A, 2B

Y. P.: 1A, 1B, 1C, 2A, 2B

A. B.: 1C, 2A, 2B

B.A.D.: 2A, 2B

C.S.: 2A, 2B

R.E.B.: 2A, 2B

A.V.: 2A, 2B

A.B.B.: 1C, 2A, 2B

J.G.: 2A, 2B

A.F.: 2A, 2B

S.K.K.: 2A, 2B

A.M.L.: 2A, 2B

M.S.: 1A, 1B, 1C, 2A, 2B

A.B.: 1A, 1B, 1C, 2A, 2B

## Disclosures

**Ethical Compliance Statement**: The authors confirm that the approval of an institutional review board was not required for this work. The subject has provided written informed consent to publish their case, including the publication of images and details of their medical history. We confirm that we have read the Journal's position on issues involved in ethical publication and affirm that this work is consistent with those guidelines.

**Funding Sources and Conflict of Interest**: No specific funding was received for this work. The authors declare no conflicts of interest relevant to this work.

**Financial Disclosures for the Previous 12 Months**: The authors declare no additional disclosures to report. AL is a consultant to Abbott, Boston Scientific, Insightec, Medtronic and Functional Neuromodulation (Scientific Director). AF received consultancy fees from Abbie, Abbott, Boston Scientific, Dompe Farmaceutici, Ipsen, Iota, and Syneos Health. Advisory Boards: Abbvie, Boston Scientific, Ceregate and Ipsen. Honoraria: Abbvie, Abbott, American Academy of Neurology, Boston Scientific, Brainlab, Ipsen, Medtronic, Merz, Movement Disorders Society, Sunovion, Paladin Labs, UCB, Sunovion. Grants: Abbvie, Boston Scientific, Dystonia Medical Research Foundation, University of Toronto, Michael J Fox Foundation, Medtronic, MSA coalition, Praxis, ES. Royalties: Springer. CS has been receiving fellowship grants from Michael and Amira Dan Foundation and Turkish Neurosurgical Society. SKK receives honoraria, consulting, and/or speaker fees from Abbott, Boston Scientific, inBrain, Medtronic, Novo Nordisk, Parkinson Canada, and Movement Disorders Society; and research support from Parkinson Canada, CIHR, MJFF, FUS Foundation, MitoO2, Toronto Western Hospital Foundation, Weston Foundation, and RR Tasker Chair in Stereotactic and Functional Neurosurgery. KY received a consulting fee from Insightec. Other authors have no disclosures to report.

## Supporting information


**Data S1.** Supplementary materials: Additional methods for the manuscript “Cerebellar Dentate Cavernoma Enlargement and Tremor Emergence: Longitudinal Neuroimaging Analysis of Case Report and Literature Review”.

## Data Availability

The data that support the findings of this study are available on request from the corresponding author. The data are not publicly available due to privacy or ethical restrictions.
