# Supplementary material for: Cerebellar Dentate Cavernoma Enlargement and Tremor Emergence: Longitudinal Neuroimaging Analysis of Case Report and Literature Review
Source: Mov Disord Clin Pract. 2025 May 19;12(9):1410–4. doi: 10.1002/mdc3.70130 (PMC12481430; doi:10.1002/mdc3.70130)
Supplement: Supplementary file 1 — Data S1. Supplementary materials: Additional methods for the manuscript “Cerebellar Dentate Cavernoma Enlargement and Tremor Emergence: Longitudinal Neuroimaging Analysis of Case Report and Literature Review”. [file MDC3-12-1410-s001.docx]

**Supplementary Materials**

**Additional methods for the manuscript "Cerebellar Dentate Cavernoma Enlargement and Tremor Emergence: Longitudinal Neuroimaging Analysis of Case Report and Literature Review"**

First, manual segmentations of the cavernoma over time were completed to identify volume change that was not easily apparent via visual interpretation. (Figure 1A) The lesion was best demonstrated on MRI T2 2D Fluid-Attenuated Inversion Recovery (FLAIR) scans, thus used for segmentation. The 2008 FLAIR sequence was acquired via GE SIGNA EXCITE 1.5T scanner: repetition time (TR)=9002ms, echo time (TE)=153ms, inversion time (IT)=2200ms, matrix=256x192, and slice thickness (ST)=7mm. In 2016 and 2018, FLAIR sequences were acquired on the same scanner: TR=9002ms, TE=166ms, TI=2200ms, matrix=256x256, and ST=5mm. The 2024 FLAIR image was acquired via SIEMENS ESPREE 1.5T scanner with a TR of 9000ms, TE of 91ms, TI of 2500ms, matrix=256x224, and ST=5mm. To ensure consistent slice orientation and spatial resolution, each FLAIR scan was registered and interpolated to match the high-resolution T1-weighted MRI image from 2024. The 2024 T1 MRI was acquired via Siemens MAGNETOM Vida 3T scanner with a 1mm isotropic resolution. Before the registration, the T1-weighted and FLAIR images were skull-stripped using SynthStrip (Freesurfer v7.4.1; https://surfer.nmr.mgh.harvard.edu/fswiki) and bias-field corrected using the N4 algorithm (ANTs; https://stnava.github.io/ANTs/). (1,2) White matter segmentation was obtained from the T1-weighted image using FAST (FSL; https://fsl.fmrib.ox.ac.uk/). (3) All FLAIR images were co-registered using FLIRT (FSL; https://fsl.fmrib.ox.ac.uk/) to the T1w in two steps: an initial alignment (cost function: Normalised Mutual Information) followed by a fine-grained adjustment using BBR (https://www.brainvoyager.com/) that utilizes the white matter boundary for precise alignment. (4,5) The cavernoma was manually segmented on the registered FLAIR images at each time point via FSLeyes (FSL; https://fsl.fmrib.ox.ac.uk/), and the lesion volumes were determined using fslstats (FSL; https://fsl.fmrib.ox.ac.uk/). (Figure 1B) (6)

Second, the impact of the patient’s cavernoma on critical motor tracts was assessed via tractography. (Figure 1C) The DRTT was selected for examination because of its role in tremor pathophysiology and the cavernoma localizing to the dentate. The DRTT is a critical neural pathway in the cerebello-thalamo-cortical network crucial for motor control, coordination, and cognitive processes. (7) The DRTT originates in the dentate nucleus of the cerebellum, projects through the superior cerebellar peduncle with most fibres decussating at the red nucleus of the midbrain and terminates in the ventrolateral nucleus of the thalamus. (7) A deterministic fibre tracking tool was used to reconstruct the DRTT (DSI Studio; https://dsi-studio.labsolver.org) using high-resolution T1-weighted structural and diffusion-weighted images (DWI). (8) The DWIs were acquired using diffusion-weighted imaging (DWI) via Siemens MAGNETOM Vida 3T scanner. A 2D-EPI diffusion sequence was used with the following parameters: directions=64, b-value=1000s/mm², b0 volumes=5, isotropic resolution=2mm, TR=7100ms, TE=76ms, matrix=128×128, GRAPPA factor=2, SMS factor=2, phase encoding (PE) direction set to anterior-posterior (AP), and bipolar diffusion gradients were applied. The tensor metrics were calculated using DWI with a b-value lower than 1000 s/mm². Preprocessing included bias field correction, skull stripping, and segmentation of T1-weighted images into brain tissues, followed by alignment to standard space (MNI 2009b; https://www.bic.mni.mcgill.ca/ServicesAtlases/ICBM152NLin2009). (8,9) Diffusion data were corrected for motion and eddy currents, aligned to T1-weighted images via rigid-body transformation, and subsequently transformed into the same standard space using T1-derived transformations. For tractography, seeds corresponding to the DRTT (dentate, red nucleus, and thalamus) were placed within the predefined brain atlas from the Human Connectome Project (HCP; https://www.humanconnectome.org), which includes predefined fibre tracts. (10) Streamlines generated from these seeds were compared to atlas-defined streamlines using Hausdorff distances. The smallest distances were retained as the best matches, whereas non-matching streamlines were excluded to ensure precise delineation of the DRTT and minimize false-positive streamlines. (11)

Third, a systematic review was conducted according to PRISMA guidelines to identify cases of 1) tremor associated with 2) cavernoma localized to the 3) dentate nucleus of the cerebellum. Databases were queried from their inception to July 3, 2024, which included Embase (*n*=32), MEDLINE(*n*=11), and Scopus (*n*=15), resulting in 58 articles. After removing duplicates (*n*=23), 35 records underwent abstract screening, leading to full text screening of 5 reports but yielded no relevant articles fitting our search criteria. (Figure 2A) (12) Therefore, as the patient’s symptom profile included rest tremor, an additional literature search was conducted of 1) Parkinsonism caused by 2) cavernoma. (Figure 2B) Databases were queried from their inception to July 3, 2024, which included Embase (*n*=45), MEDLINE (*n*=13), and Scopus (*n*=31), resulting in 89 articles. After removing duplicates (*n*=41), 48 records underwent abstract screening, leading to full-text screening of 11 reports, resulting in the inclusion of 8 articles for the final analysis. (13–20)

**References**

1. Hoopes A, Mora JS, Dalca AV, Fischl B, Hoffmann M. SynthStrip: skull-stripping for any brain image. NeuroImage. 2022 Oct 15;260:119474.

2. Tustison NJ, Avants BB, Cook PA, Yuanjie Zheng, Egan A, Yushkevich PA, et al. N4ITK: Improved N3 Bias Correction. IEEE Trans Med Imaging. 2010 Jun;29(6):1310–20.

3. Zhang Y, Brady M, Smith S. Segmentation of brain MR images through a hidden Markov random field model and the expectation-maximization algorithm. IEEE Trans Med Imaging. 2001 Jan;20(1):45–57.

4. Jenkinson M, Smith S. A global optimisation method for robust affine registration of brain images. Medical Image Analysis. 2001 Jun 1;5(2):143–56.

5. Greve DN, Fischl B. Accurate and robust brain image alignment using boundary-based registration. Neuroimage. 2009 Oct 15;48(1):63–72.

6. Jenkinson M, Beckmann CF, Behrens TEJ, Woolrich MW, Smith SM. FSL. Neuroimage. 2012 Aug 15;62(2):782–90.

7. Rios-Zermeno J, Ballesteros-Herrera D, Dominguez-Vizcayno P, Carrillo-Ruiz JD, Moreno-Jimenez S. Dentate nucleus: a review and implications for dentatotomy. Acta Neurochir. 2024 May 17;166(1):219.

8. Yeh FC, Verstynen TD, Wang Y, Fernández-Miranda JC, Tseng WYI. Deterministic Diffusion Fiber Tracking Improved by Quantitative Anisotropy. Zhan W, editor. PLoS ONE. 2013 Nov 15;8(11):e80713.

9. Fonov V, Evans AC, Botteron K, Almli CR, McKinstry RC, Collins DL. Unbiased average age-appropriate atlases for pediatric studies. NeuroImage. 2011 Jan;54(1):313–27.

10. Sotiropoulos SN, Moeller S, Jbabdi S, Xu J, Andersson JL, Auerbach EJ, et al. Effects of image reconstruction on fiber orientation mapping from multichannel diffusion MRI: Reducing the noise floor using SENSE. Magnetic Resonance in Med. 2013 Dec;70(6):1682–9.

11. Yeh FC. Shape analysis of the human association pathways. Neuroimage. 2020 Dec;223:117329.

12. The PRISMA 2020 statement: an updated guideline for reporting systematic reviews - PubMed [Internet]. [cited 2025 Jan 10]. Available from: https://pubmed.ncbi.nlm.nih.gov/33782057/

13. Vhora S, Kobayashi S, Okudera H. Pineal cavernous angioma presenting with Parkinsonism. 2001;

14. Ertan S, Benbir G, Tanriverdi T, Alver I, Uzan M. Parkinsonism caused by cavernoma located in basal ganglion. Parkinsonism & Related Disorders. 2005 Dec;11(8):517–9.

15. Li ST, Zhong J. Surgery for mesencephalic cavernoma: case report. Surgical Neurology. 2007 Apr;67(4):413–7.

16. Alp R, Alp Sİ, Üre H. Cavernous Hemangioma: A Rare Cause for Secondary Parkinsonism: A Case Report. International Journal of Neuroscience. 2009 Jan;119(11):2112–7.

17. Ghaemi K, Krauss JK, Nakamura M. Hemiparkinsonism due to a pontomesencephalic cavernoma: improvement after resection: Case report. PED. 2009 Aug;4(2):143–6.

18. Dall’aglio Rocha CE, Nazaré Oliveira FD, Meguins LC. Hemiparkinsonism associated with mesencephalic cavernoma: case report and review of literature. Arq Bras Neurocir. 2011 Dec;30(04):194–8.

19. Aydin S, Esen Aydin A, Yuksel O, Tanriverdi T. Secondary Parkinsonism in a Patient With a Cerebral Cavernous Hemangioma Treated With Stereotactic Radiosurgery. Cureus [Internet]. 2021 Mar 26 [cited 2024 Aug 15]; Available from: https://www.cureus.com/articles/53142-secondary-parkinsonism-in-a-patient-with-a-cerebral-cavernous-hemangioma-treated-with-stereotactic-radiosurgery

20. Modreanu RM, Buhmann C, Hauptmann B. Nine-years follow-up of cavernoma located in basal ganglia mimicking Parkinson’s disease. Clinical Neurology and Neurosurgery. 2020 Mar 1;190:105664.
